# Kyasanur Forest Disease Virus Infection in Mice Is Associated with Higher Morbidity and Mortality than Infection with the Closely Related Alkhurma Hemorrhagic Fever Virus

**DOI:** 10.1371/journal.pone.0100301

**Published:** 2014-06-20

**Authors:** Kimberly A. Dodd, Brian H. Bird, Megan E. B. Jones, Stuart T. Nichol, Christina F. Spiropoulou

**Affiliations:** 1 Viral Special Pathogens Branch, Division of High Consequence Pathogens and Pathology, Centers for Disease Control and Prevention, Atlanta, Georgia, United States of America; 2 University of California, Davis, School of Veterinary Medicine, Davis, California, United States of America; 3 Department of Pathology, College of Veterinary Medicine, University of Georgia, Athens, Georgia, United States of America; National Institutes of Health. National Institute of Allergy and Infectious Diseases, Division of Clinical Research, United States of America

## Abstract

**Background:**

Kyasanur Forest disease virus (KFDV) and Alkhurma hemorrhagic fever virus (AHFV) are closely related members of the Flavivirus genus and are important causes of human disease in India and the Arabian Peninsula, respectively. Despite high genetic similarity, the viruses have distinctly different host ranges and ecologies. Human cases of KFDV or AHFV develop a spectrum of disease syndromes ranging from liver pathology to neurologic disease. Case reports suggest KFDV is more commonly associated with hepatic and gastrointestinal manifestations whereas AHFV is more commonly associated with neurologic disease.

**Methodology/Principal Findings:**

Inoculation of three immunocompetent laboratory mouse strains revealed that KFDV was consistently more lethal than AHFV. In subsequent studies utilizing C57BL/6J mice, we demonstrated that KFDV infection was associated with higher viral loads and significantly higher mortality. KFDV-infected mice rapidly developed more severe disease than AHFV-infected mice, as evidenced by significant abnormalities on clinical chemistry panels and more severe pathology in the brain and gastrointestinal tract.

**Conclusions/Significance:**

Infections of C57BL/6J mice with KFDV or AHFV resulted in clinical disease syndromes that closely approximate the diseases seen in human cases. Despite high genetic similarity, there were clear differences in survival, viral kinetics, clinical chemistry data and histology. These results suggest that distinct mouse models for AHFV and KFDV are necessary in order to gain a better understanding of the unique pathogenesis of each virus, as well as to provide platforms for testing promising vaccines and therapeutics.

## Introduction

Alkhurma hemorrhagic fever virus (AHFV) and Kyasanur Forest disease virus (KFDV) are closely related members of the genus Flavivirus [family *Flaviviridae*]. The genus is subdivided by vector into three groups: mosquito-borne, tick-borne, and a group including flaviviruses with no known vector. Several tick-borne flaviviruses (AHFV, KFDV, Omsk hemorrhagic fever virus (OHFV), Powassan virus, and tick-borne encephalitis virus), as well as a number of mosquito-borne flaviviruses (West Nile virus, Dengue virus, Yellow fever virus, Japanese encephalitis virus, etc.) are important human pathogens.

The closely related AHFV and KFDV cause similar disease syndromes in people, marked by sudden onset fever, myalgia and arthralgia. In severe cases, sequelae can include encephalitis and/or a hemorrhagic syndrome, with the latter defined by petechiae, epistaxis, bleeding from gums, hematemesis, melena and hematochezia [1]–[5]. Most commonly in AHFV cases, neurologic clinical signs have been described, including tremors, seizures, neck rigidity, confusion, convulsions or coma [1]–[5]. In contrast, a review of fatal human cases of KFDV suggested that gastrointestinal hemorrhage is the most common finding [4]. The overall case fatality of KFDV infection is estimated to be 1–2% of the 400–500 cases reported annually, and a recent outbreak suggested AHFV mortality is similar to KFDV (<2%) [2]. Previously, reports of AHFV were associated with a higher case fatality ratio of 25% [5] that indicated a failure to identify asymptomatic or mild cases initially. More recently, the number of human AHFV infections documented annually is generally less than 100 [6]. Beyond supportive care, there is no specific treatment for either AHFV or KFDV infection.

Despite their high genetic similarity (>92% by nucleotide) and the similar clinical syndromes they cause in humans, AHFV and KFDV diverged more than 700 years ago [7] and have since maintained distinct geographic ranges, primarily in Saudi Arabia and India, respectively. However, the viruses were only first identified during the second half of the 20^th^ century. In 1957, KFDV was recognized as the cause of human disease in the Shimoga district of India and concurrent massive deaths of nonhuman primates in the Kyasanur Forest. KFDV human cases remain isolated to regions within Karnataka State. Almost 40 years later in 1994, AHFV was isolated from a fatally infected butcher in Makkah, Saudi Arabia. Since then, AHFV cases have been confirmed in Jeddah, Jizan, and Najran in Saudi Arabia and most recently across the Red Sea, near the Egypt-Sudan border in 2010 [8].

Risk factors associated with KFDV infection include activities in the Kyasanur Forest region, handling ill or dead primates, exposure to ticks (primarily *Haemaphysalis spp*), or through laboratory infections [1]. Human AHFV infections have also been associated with tick bites, and AHFV has been isolated from an *Ornithodoros* tick in Jeddah [9], and *Ornithodoros savignyi* and *Hyalomma dromedarii* ticks in Najran [10]. However, another common risk factor for AHFV infection appears to be close contact with domestic animals, particularly sheep and camels [3], although no disease has been reported in these animals.

A KFDV vaccine is available, however, the efficacy of this vaccine is unclear [1], and hundreds of human KFDV cases reported every year. Recently, an upsurge of cases in 2012 has been attributed to a shortage of this vaccine and the subsequent failure to provide booster vaccinations to individuals at high risk [11]. The increasing number of KFDV cases, coupled with AHFV range expansion into east Africa, highlights the need for efficacious vaccines and development of antiviral drugs. Here we describe the development of lethal disease models of KFDV and AHFV infection for this purpose. Previously, KFDV infection was described in several species, including laboratory rodents and wild animals (reviewed in [12]). However, this work describes the first model of AHFV infection in the mouse model, and therefore provides the first opportunity to directly compare the relative pathogenesis of these genomically similar, yet ecologically distinct viruses.

## Materials and Methods

### Ethics statement

Animal procedures in this study complied with institutional guidelines, the US Department of Agriculture Animal Welfare Act, and the National Institutes of Health Guide for the Care and Use of Laboratory Animals. All procedures were approved by the Centers for Disease Control and Prevention (CDC) Institutional Animal Care and Use Committee (IACUC).

### Biosafety

All work with infectious KFDV and AHFV was completed in a biosafety level (BSL)-4 laboratory at the Centers for Disease Control and Prevention (CDC, Atlanta, GA, USA). All animals were housed within the BSL-4 laboratories in microisolator pans in HEPA filtration racks, following standard barrier techniques. All laboratorians and animal handlers adhered to international biosafety practices appropriate for BSL-4, strictly following infection control practices to prevent cross-contamination between individual animals.

### Animals and husbandry

Female 6–8 week old C3H, A/J and C57BL/6J mice were obtained from a commercial vendor (Jackson Laboratory). Mice were housed in groups of 5, and supplied a commercially available mouse chow and water *ad libitum*. After infection, each animal was observed a minimum of once per day, and its health assessed and scored by experienced CDC veterinarians or animal health technicians. Animals were euthanized if found in acute distress or moribund, or if they scored greater than 10 on a pre-determined clinical illness scoring algorithm: (2 points) hunched posture, ruffled coat or huddling; (3 points) ataxia, circling, paresis, or tremors; (5 points) apparent anemia (pale mucous membranes), or difficulty breathing.

### Viruses

KFDV strain P9605 was originally isolated in 1957 from serum of a human patient in Shigga, Karnataka. Wild-type KFDV (strain P9605) and AHFV (strain 200300001) were grown from stocks in the CDC Viral Special Pathogens Branch reference collection. Viruses were propagated in VeroE6 cells with Dulbecco's modified Eagle's medium (DMEM) supplemented with 5% fetal bovine serum (FBS) and penicillin-streptomycin (Invitrogen). Titers of all viral stocks were determined as tissue culture infective dose 50 (TCID_50_) on VeroE6 cells and visualized by indirect fluorescent-antibody assay (IFA) using anti-KFDV hyperimmune mouse ascitic fluid (HMAF) primary antibody.

### Animal infections

In the first animal experiment, inbred mouse strains were evaluated for use as pathogenesis models of KFDV and AHFV infections. Three common immunocompetent laboratory mouse strains (C3H, C57BL/6J, and A/J) were inoculated in groups of 10 with either 1.0×10^5^ TCID_50_ KFDV or 1.0×10^5^ AHFV TCID_50_ subcutaneously (sc) in a volume of 100 µL sterile DMEM. Mock-infected controls were inoculated with 100 µL sterile DMEM. Animals were evaluated twice daily for 28 days following infection.

In a follow-up experiment, a 50% lethal dose (LD50) study was undertaken to determine the dose of virus required to cause mortality in 50% of infected C57BL/6J mice. All mice were implanted with an identification chip for noninvasive measurement of body temperature. Mice in groups of 5 were infected sc with AHFV or KFDV at the following doses: 100,000; 10,000; 1,000; 100; 10; or 1 TCID_50_.

A serial euthanasia study was next undertaken utilizing the C57BL/6J mouse model to compare the kinetics of viral spread, clinical parameters of disease progression, innate immune response and virus-associated pathology of mice infected with 1×10^5^ TCID_50_ of AHFV or 1×10^5^ TCID_50_ KFDV. On 1, 4, 6, and 7 days post-infection (dpi), 5 AHFV- and 5 KFDV-infected mice were anesthetized with isofluorane, terminally bled and perfused with PBS. Whole blood was taken for complete blood counts (CBC) and a metabolic blood chemistry panel. Samples of liver, spleen, kidney, gastrointestinal tract (GIT) and brain were taken for quantitation of virus load and histology.

### Hematological parameters

Whole blood was collected under general anesthesia by intracardiac puncture into either EDTA-coated or heparin-coated vacutainer tubes. CBCs were performed using the Hematrue blood analyzer (HESKA). Blood chemistry profiles were obtained from heparinized whole blood samples using the Piccolo point of care chemistry analyzer (Abaxis).

### Total RNA extraction

Liver, spleen, kidney, GIT, brain and blood specimens were collected on days 1, 4, 6 and 7 post-infection, and from animals reaching experimental end points. RNA was extracted using MagMax Total RNA Isolation kit (Ambion). Approximately 100 mg tissue samples were placed directly in lysis buffer and homogenized using a high throughput tissue grinder (GenoGrinder2000). Homogenates were extracted using the MagMax Express-96 Magnetic Particle Processor (Ambion) according to manufacturer's directions including a DNase treatment step. Approximately 50 mL of whole blood was added directly to lysis buffer with isopropanol and extracted using the MagMax Express-24 Magnetic Particle Processor (Ambion) following manufacturer's protocol.

### Quantitative reverse-transcription PCR (qRT-PCR) for viral RNA quantification

AHFV and KFDV RNA were detected using primers and probe targeting a conserved region of the NS3 protein (forward primer: 5′-ATGAGTGAGGAAAGGGCCAT-3′; reverse primer: 5′-CTCATACTCTGTTATCCAGTC-3′; probe: 5′-6FAM-ACGGAGAGTGGAGAGAAGGCTT-3′). For each viral genome detection reaction, 2.5 µL of total RNA (approximately 0.5 µg RNA) was added to a one-step qRT-PCR reaction (SuperScript III Platinum One-Step qRT-PCR kit, Invitrogen) and run using the ABI 7500 or Viia7 Real-Time PCR systems (Applied Biosystems). RNA genome equivalents in infected blood and tissue specimens were obtained using standard curves generated by serial dilutions of the same known-titer stocks of AHFV and KFDV used for infection. The results of all qRT-PCR runs were normalized to endogenous mouse-specific controls (glyceraldehyde 3-phosphate dehydrogenase (GAPDH), Invitrogen) following the manufacturer's recommended protocols to account for sample-to-sample variation.

### Antiviral assays

Mouse antiviral Response quantitative PCR arrays (Qiagen SAMM-122Z) were used to determine relative gene expression of a select panel of 84 antiviral genes in mice infected with AHFV or KFDV, relative to mock infected mice. Assays were run on brain samples from 3 AHFV-infected mice, 3 KFDV-infected mice, and 3 mock-infected mice euthanized on 1 and 4 dpi. For each sample, cDNA was synthesized from 0.8–1.0 µg of RNA using the RT^2^ first strand kit (SABioscience). Arrays were run on an ABI 7500 using RT^2^ SYBR Green/ROX PCR master mix according to manufacturer's instructions (SABioscience).

### Histology

At the time of collection, liver, spleen, brain, intestine, and kidney tissue specimens were fixed by immersion in 10% neutral buffered formalin for 7 days and gamma-irradiated (2.0×10^6^ RAD) prior to processing. Tissues were paraffin-embedded following routine methods, sectioned at approximately 4 micrometers, and stained with hematoxylin and eosin (H&E) for histological examination.

### Statistical analyses

All analyses were completed using the PRISM v5.0 program (Graphpad). For each mouse strain, differences between AHFV and KFDV survivor curves were evaluated using the Log-rank (Mantel-Cox) test. Potentially significant differences between AHFV and KFDV viral loads in tissues were evaluated using a two-way ANOVA with Bonferroni post-tests for multiple comparisons. For the complete blood counts and clinical chemistry data, significant differences between infected and sham-infected animals at each time point were analyzed using a one-way analysis of variance (ANOVA) with Dunnett's adjustment for multiple comparisons (*p<0.05; **p<0.01, ***p<0.001). For the antiviral array analysis, the mean value for each gene was calculated from replicate tissue samples using the threshold cycle (CT) method and normalized to the average values for five housekeeping genes (Gus-β, Hprt, HSP-90AB1, GAPDH, and β-actin genes). The *P* values were calculated using Student's t test (SABioscience) for each gene in the AHFV and KFDV-infected groups.

## Results

### KFDV is more virulent than AHFV in 3 inbred mouse strains

**Figure 1:**
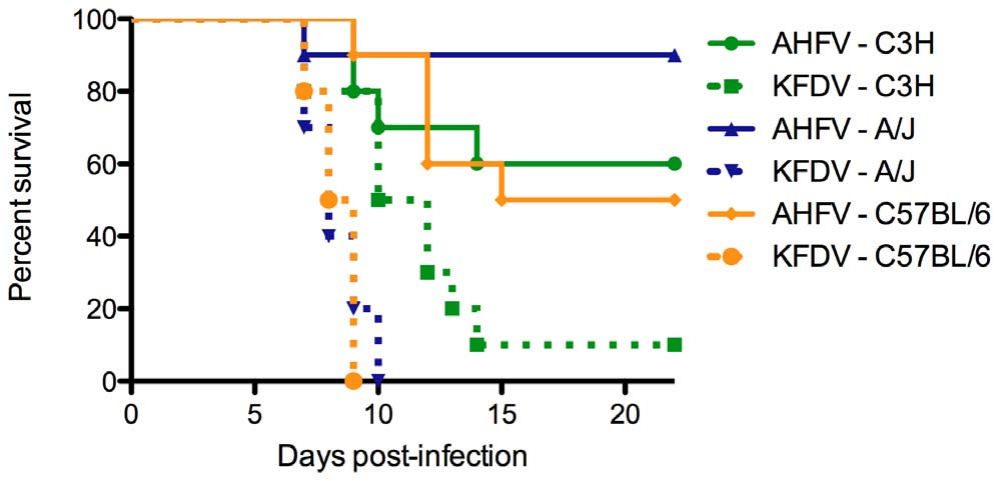
KFDV is more virulent than AHFV in 3 immunocompetent mouse strains. C3H, A/J and C57BL/6J mice were inoculated subcutaneously with either 1×10^5^ TCID_50_ AHFV or 1×10^5^ TCID_50_ KFDV (n = 10/group) and monitored for 28 days. Regardless of strain, KFDV infection resulted in higher mortality and more severe disease than AHFV.

Three common laboratory mouse strains were infected via the subcutaneous route with either 10^5^ TCID_50_ KFDV or 10^5^ TCID_50_ AHFV sc. KFDV-infected mice displayed signs of progressive illness including hunched posture, ruffled fur and lethargy starting 6 dpi with tremors developing in some mice prior to death. Onset of similar clinical disease occurred later in AHFV-infected mice, beginning on 9 dpi, with indications of neurologic disease including hind-limb paralysis, ataxia and/or tremors. At no point during the course of infection did KFDV- or AHFV-infected mice become febrile; in both groups, body temperature did not significantly differ from mock-infected mice. Mortality was significantly higher in all mice infected with KFDV relative to AHFV, regardless of strain: C57BL/6: 100% vs. 50% (p<0.0001), C3H: 90% vs. 60% (p<0.05), A/J 100% vs. 10% (p<0.0001) (Figure 1). In the subsequent LD_50_ assay in C57BL/6J mice, the LD_50_ of KFDV (<1 TCID_50_) was significantly lower than that of AHFV (>10^5^ TCID_50_) (Table 1).

**Table 1:** Survival data for C57BL/6 mice following infection with KFDV or AHFV sc.

Dose (TCID_50_)	1	10	100	1000	10000	100000
**KFDV**	2/5	0/5	0/5	0/5	0/5	0/5
**AHFV**	5/5	5/5	4/5	5/5	5/5	3/5

Values indicate the number of mice (out of 5) that survived infection with KFDV or AHFV as of 30 dpi.

### Peak viral RNA loads were found in the tissues of KFDV-infected mice earlier than AHFV-infected C57BL/6J mice

**Figure 2:**
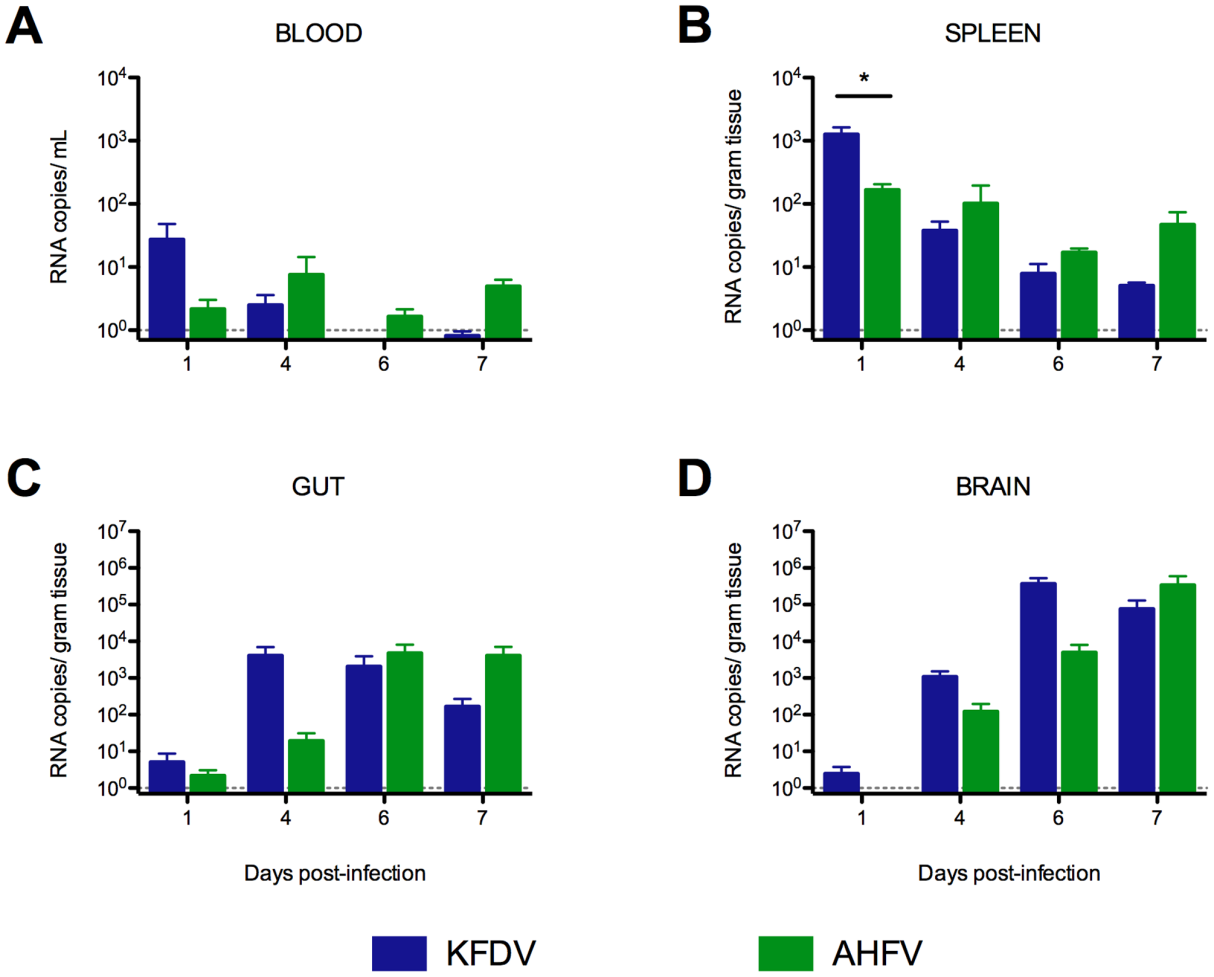
KFDV-infected mice have higher viral RNA loads than AHFV-infected mice early in infection. Viral RNA loads in the (A) blood, (B) spleen, (C) gastrointestinal tract and (D) brain were compared between KFDV- and AHFV-infected mice on 1, 4, 6 and 7 dpi. Asterisks indicate significant differences between KFDV- and AHFV-infected mice (n = 5; p<0.05). The gray dotted line denotes limit of detection of the assay.

KFDV-infected mice had higher viremia 1 day post-infection (dpi), but blood from AHFV-infected mice had vRNA present through 7 dpi (Figure 2A). The highest vRNA titers in the spleens were found 1 dpi, suggesting the spleen is an early site for virus amplification for both viruses (Figure 2B). Very low amounts of vRNA were found in the liver and kidney of mice in both groups throughout the course of infection (data not shown). KFDV-infected mice had high vRNA loads in the gastrointestinal tract (Figure 2C) and brain (Figure 2D) beginning 4 dpi. Two days later, AHFV levels in the GIT were equivalent to those of KFDV-infected mice. However, the brains of KFDV-infected mice had significantly higher vRNA loads than AHFV-infected until 7 dpi.

### KFDV- and AHFV-infected mice developed transient lymphopenia

**Figure 3:**
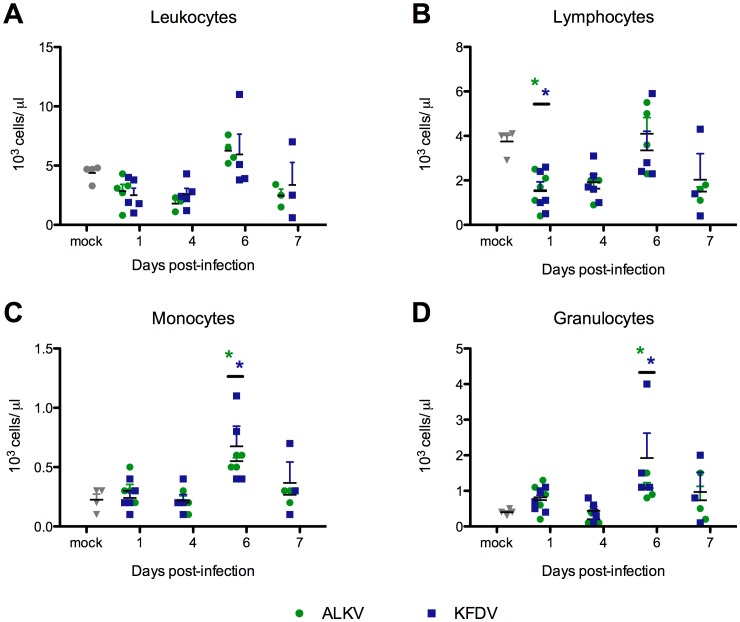
KFDV and AHFV-infected mice developed significant lymphopenia relative to control mice. Complete blood counts were run on KFDV-, AHFV- and mock-infected mice on 1, 4, 6, and 7 dpi. (A) Leukocyte, (B) lymphocyte, (C) monocyte and (D) granulocyte counts are displayed. Asterisks indicate significant differences between KFDV-infected mice (blue) or AHFV-infected mice (green) relative to mock-infected animals (p<0.05). On each day, data was collected from a minimum of 3 and a maximum of 5 mice per group.

CBC data indicated that both AHFV- and KFDV-infected mice developed marked lymphopenia that was significant 1 dpi (Figure 3B). By 6 dpi, mice in both groups had significant monocytosis (Figure 3C) and neutrophilia (Figure 3D). There were no other alterations in the CBC data, and no significant differences between KFDV- and AHFV-infected mice.

### KFDV-infected mice, but not AHFV-infected mice, developed elevated liver enzymes, azotemia, hypoalbuminemia and hypoglycemia late in infection

**Figure 4:**
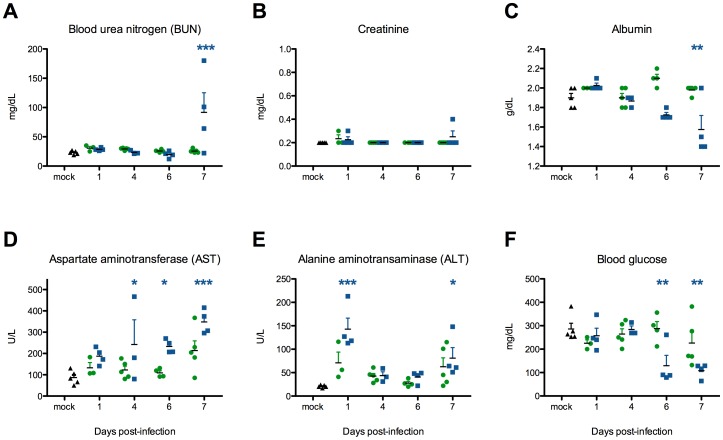
KFDV infection and not AHFV infection resulted in significant biochemical changes, particularly at late time-points post-infection. KFDV infection was associated with (A) elevated BUN, (B) slightly increased creatinine, (C) elevated ALT, (D) elevated AST, (E) hypoalbuminemia and (F) hypoglycemia. Asterisks indicate significant differences between KFDV-infected mice (blue) relative to mock-infected animals (p<0.05). No alterations in clinical chemistry values were significant in AHFV-infected mice (green). On each day, data was collected from a minimum of 3 and a maximum of 5 mice per group.

Chemistry data from KFDV-infected mice indicated significant deviations from normal values. KFDV-infected mice had increased blood-urea nitrogen (BUN) 7 dpi (Figure 4A) and normal creatinine (Figure 4B), driving an increased BUN/creatinine ratio. Liver enzymes were elevated in KFDV-infected mice: ALT on 1 and 7 dpi (Figure 4C), and AST from 4 dpi through the end of the infection (Figure 4D). During the late stages of the disease, hypoalbuminemia (Figure 4E) and hypoglycemia (Figure 4F) were apparent in KFDV mice. In contrast, all chemistry values from AHFV-infected mice were indistinguishable from mock-infected mice.

### Antiviral gene expression was elevated in brains of AHFV- and KFDV-infected mice

**Figure 5:**
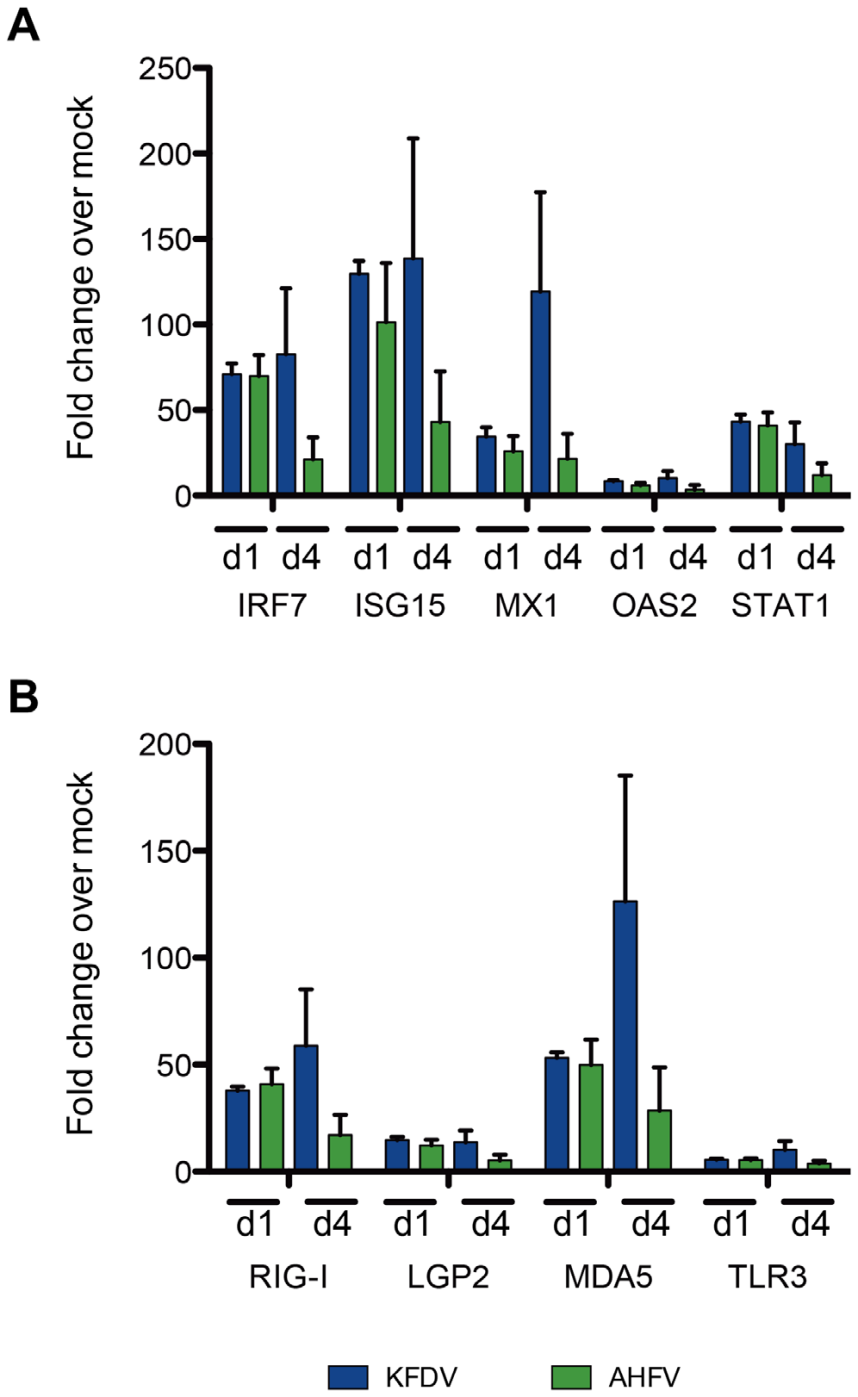
KFDV- and AHFV-infected mice mount an early innate response to infection in the CNS. In the brains of mice infected with KFDV or AHFV, there was significant upregulation of (A) interferon-stimulated genes and (B) pathogen recognition receptors (PRRs). There were not significant differences in gene expression between mice infected with AHFV and those infected with KFDV (n = 5; p>0.05).

In order to address the possibility that the higher viral loads and more rapid virus dissemination in KFDV-infected mice relative to AHFV-infected mice were due to reduced antiviral immune responses, we compared antiviral gene expression between groups. We focused on the immune response in the CNS because it is a major target of viral infection, and KFDV-infected mice had viral RNA present in the CNS earlier and at higher levels than AHFV-infected mice. We therefore compared the interferon-stimulated responses in the brains of mice 1 and 4 dpi, before virus-mediated pathology was apparent in the brain. Relative to mock-infected mice, both groups of infected mice demonstrated significant upregulation of interferon stimulated genes (ISGs) including IRF7, ISG15, Mx1, OAS and STAT1 on 1 and 4 dpi (Figure 5A). AHFV- and KFDV-infected mice also had significant upregulation of dsRNA sensors, including the cytosolic helicases RIG-I, MDA5 and LGP2, as well as TLR3 (Figure 5B). There were no significant differences in antiviral gene expression between AHFV- and KFDV-infected mice.

### Histopathologic lesions occurred earlier and were more severe in KFDV-infected mice than in AHFV-infected mice

**Figure 6:**
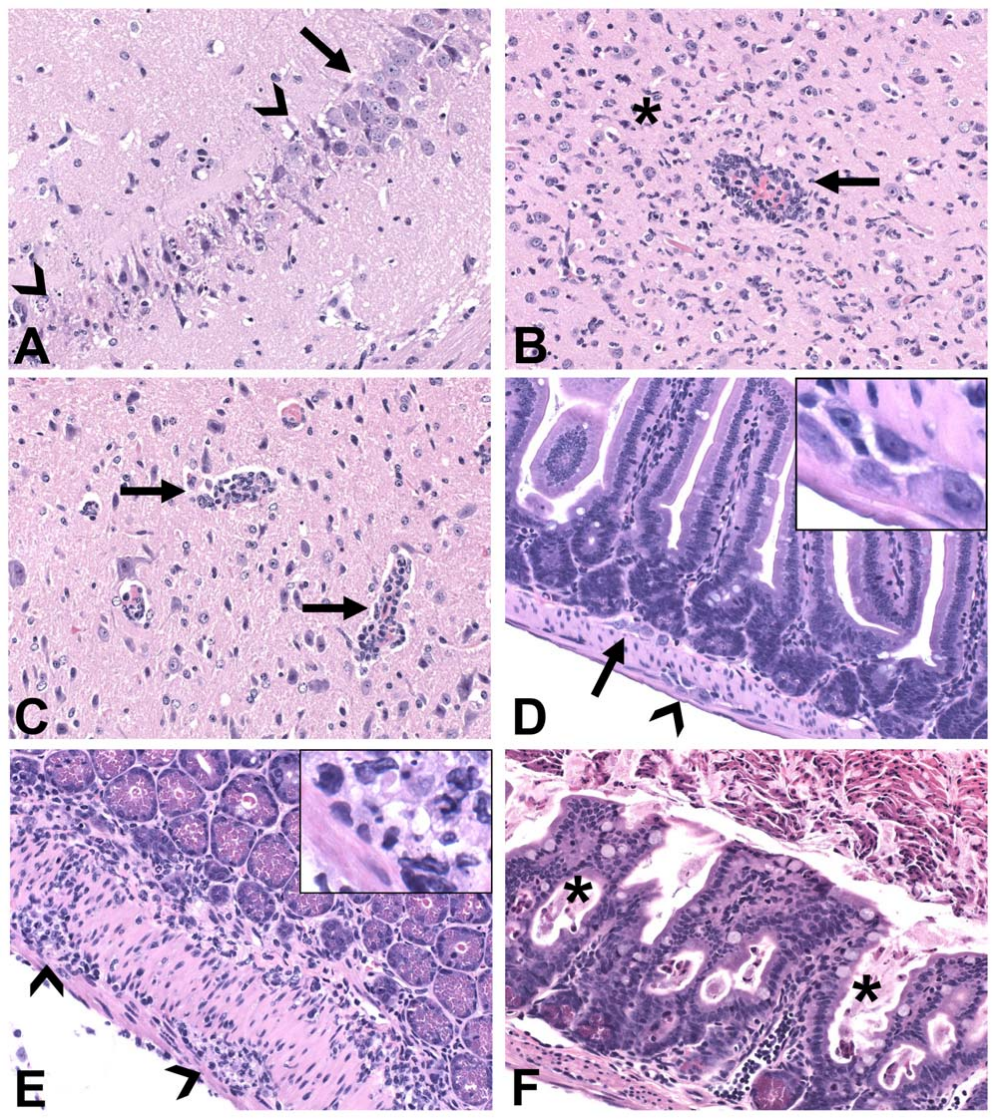
More severe histopathologic lesions were apparent in the brains and gastrointestinal tracts of KFDV-infected mice than AHFV-infected mice. (A) Brain, hippocampus, KFDV-infected mouse, 6 dpi; acute necrosis and loss of hippocampal neurons (segment delineated by arrowheads), with adjacent, relatively unaffected neurons (arrow). (B) Brainstem, KFDV-infected mouse, 6 dpi; severe meningoencephalitis with perivascular cuffing (arrow) and widespread gliosis in the adjacent neuropil (asterisk). (C) Brainstem, AHFV-infected mouse, 7 dpi; mild meningoencephalitis characterized by perivascular cuffing (arrows). (D) Small intestine, mock-inoculated mouse; normal tissue section demonstrating anatomic location of submucosal (arrow) and myenteric (arrowhead) nerve plexi and normal intestinal villi; inset shows higher magnification of neuron cell bodies in plexus. (E) Small intestine, KFDV-infected mouse, 7 dpi; moderate, predominantly histiocytic, inflammatory infiltrate in submucosa and muscularis, involving and disrupting myenteric plexi (arrowheads). Inset shows higher magnification of infiltrate and cell debris in plexus. (F) Small intestine, KFDV-infected mouse, 7 dpi; intestinal crypts are dilated and filled with necrotic cells (asterisks), intestinal lumen contains abundant cellular debris (top of image), and villi are blunted and fused. All H&E images, original magnification 200x.

The earliest histopathologic lesions consisted of multifocal splenic fibrin thrombi at 1 dpi in three of five KFDV-infected mice. No other splenic lesions were identified in any virus- or mock-infected mice on any day. Early brain lesions consisted of very mild perivascular cuffing in one of five KFDV-infected mice at 4 dpi. By 6 and 7 dpi, all KFDV-infected mice exhibited moderate to severe meningoencephalitis with lymphocytic and histiocytic perivascular cuffing, acute neuronal necrosis, microgliosis, and glial nodules (Figure 6A and 6B). Areas of gliosis and perivascular cuffs sometimes also contained neutrophilic infiltrate and karyorrhectic cell debris. Lesions were most consistently present in the cerebral cortex, thalamus, midbrain, and brainstem, but the olfactory bulb, hippocampus, and cerebellum were also variably involved. Brain lesions in AHFV-infected mice were milder, affected fewer individuals, and were delayed in onset compared to KFDV-infected mice. Mild meningitis and perivascular cuffing first appeared at 6 dpi in only one of five AHFV mice (Figure 6C). At 7 dpi, one of five AHFV-infected mice had meningoencephalitis of similar character and severity to lesions in 6–7 dpi KFDV-infected mice, while two mice exhibited only mild perivascular cuffing and two remained unaffected.

Small intestine was available for histologic examination in both infected groups from 6 and 7 dpi, and from mock-inoculated mice. Small intestinal lesions were most significant in KFDV-infected mice, and consisted of histiocytic, lymphocytic, and variably neutrophilic infiltrate in the submucosa and muscularis. This inflammation often involved, obscured, and disrupted submucosal and myenteric nerve plexi (Figure 6E; normal plexi for comparison, mock-infected mice, Figure 6D). KFDV-infected mice also had evidence of an acute, necrotizing process characterized by intestinal crypt dilation and necrosis, and accumulation of abundant cell debris in the intestinal lumen (Figure 6F). Enteritis in AHFV-infected mice was less severe than that in KFDV mice, and consisted only of mixed inflammation in the submucosa and muscularis. Lesions were minimal at 6 dpi and mild at 7 dpi, affecting 3 and 5 mice, respectively. As in KFDV mice, inflammation sometimes centered on submucosal and myenteric plexi. In the liver of KDFV-infected mice, there was mild microvesicular hepatocellular vacuolation (lipidosis, consistent with inanition) on days 6 and 7. No significant lesions were identified in kidneys from any group, or in any tissues from mock-infected mice.

## Discussion

Tickborne flaviviruses, including OHFV, TBEV, AHFV and KFDV, are important human pathogens in certain regions of the world. AHFV was identified almost 40 years after the initial description of KFDV, and the remarkable genetic similarity led to the initial classification of AHFV as a subtype of KFDV. However, their different geographic ranges, ecologies, and recent phylogenetic analyses suggest that these viruses diverged hundreds of years ago [2], [7]. AHFV, found originally in Saudi Arabia, has since been found repeatedly in Egypt in close association with camel markets [5], [13]. In contrast, all the known KFDV cases occur with a small region surrounding the Kyasanur Forest in southwest India. Given these apparent differences in ecology, public health impact and host range we evaluated the viruses for differences in clinical disease and mortality, viral kinetics, clinical parameters and pathology in mice.

In an initial study, three commonly used laboratory mouse strains were tested for susceptibility to KFDV and AHFV. C57BL/6, C3H and A/J mice are all commonly used and immunocompetent mouse strains; they differ immunologically in their MHC H2 haplotypes. In all three strains, KFDV infections resulted in a more rapid and severe disease than seen with AHFV-infected mice. Of the mice infected with KFDV, 90–100% succumbed to infection, as compared 10–50% mortality seen following AHFV infection. It is important to note that mice infected with KFDV or AHFV received the same dose of virus, despite the differences in LD50 between groups, in order to directly compare gross differences in pathogenesis between the two viruses. Future studies to compare more subtle differences in pathogenesis might provide valuable information by utilizing virus doses with equivalent LD50s.

KFDV and AHFV have much lower case fatality rates in humans, however, clinical disease in the mouse models, and particularly the C57BL/6 mice, were strikingly similar to case reports of severe human disease. KFDV-infected mice consistently demonstrated gross anatomical signs of gastrointestinal hemorrhage, which correlates with the primary autopsy findings in a broad evaluation of 100 human KFDV cases [4], [6]. AHFV-infected mice had clinical indications of neurologic disease, including partial paralysis and tremors, as has been described in AHFV human infections [2], [3], [7]. KFDV and AHFV disease in the mouse model differed from published descriptions of human disease; mice did not become febrile or develop overt symptoms of biphasic or delayed-onset disease.

Both AHFV and KFDV patients have consistently been described as having CBC abnormalities including lymphopenia [1], [2], [4], [8], [14]–[16], as was seen in KFDV- and AHFV-infected mice. Transient lymphopenia is a common finding following viral infection and has been shown to be associated with an early Type I interferon response [17]. Reports of both KFDV and AHFV human cases have described significant hypoalbuminemia, elevated blood urea nitrogen (BUN) and liver transaminases [4], [5], [9], [18]. In the mouse model, KFDV-infected mice, but not AHFV-infected mice had values significantly greater than mock-infected mice, which may explain the more rapid and severe course of disease in KFDV infection.

KFDV-infected mice succumbed to infection within 7–9 days. Hypoglycemia and presumptive lipidosis in these mice suggest anorexia as a prelude to the development of clinical disease. In contrast, approximately one-half of AHFV-infected mice developed fatal disease between 9 and 15 dpi characterized by signs of neurologic involvement, while the remaining half survived with no indication of clinical signs. In both groups, rapid viral dissemination to the spleen occurs within 24 hours after infection, although viral loads in AHFV-infected mice were one log lower than KFDV-infected mice. High levels of virus replication and associated evidence of progressive mild to moderate meningoencephalitis was evident as early as 4–6 dpi in KFDV-infected mice but lagged by 2–3 days in AHFV-infected mice. Viral replication was apparent in the small intestine of KFDV-infected mice at the same time, and by 6 dpi, there was histologic evidence of an acute necrotizing process. Significant elevation in BUN of KFDV-infected mice and an elevated BUN/creatinine ratio, coupled with the hypoalbuminemia, are consistent with GI hemorrhage. AHFV loads were significantly lower in the GIT through 6 dpi, with minimal enteritis evident and there were no abnormalities in the clinical chemistry data indicative of GI hemorrhage. In both KFDV- and AHFV-infected mice, intestinal lesions included inflammation of enteric nerve plexi, associated with neuronal degeneration and depletion. This finding has also been reported in mouse models of West Nile virus [10], [19]. 

Both viruses stimulated increased antiviral gene expression relative to mock-infected control mice and there were no significant differences between AHFV and KFDV in any of the 86 antiviral genes evaluated. These results suggested that the viruses stimulate similar responses in the CNS by triggering a type I interferon response as evidenced by upregulation of interferon-stimulated genes Mx1, OAS, ISG15, IRF7 and STAT1. Increased expression of RIG-I and MDA5 suggested that AHFV and KFDV could be recognized by one or both of the cytosolic helicases, as has been demonstrated for JEV, WNV, DENV and possibly YFV (reviewed in [3], [20]). Similarly, upregulation of TLR3 suggests that dsRNA replication intermediates might be recognized in the endosome as has been postulated for WNV and DENV [20].

In summary, KFDV infected mice displayed higher morbidity and mortality than AHFV-infected mice in the C57BL/6J mouse model. KFDV-infected mice uniformly succumbed to disease 7–9 days post-infection, following a rapid disease progression characterized by high viral loads, significant clinical chemistry abnormalities and marked pathology in the gastrointestinal tract and brain. In contrast, approximately 50% of AHFV-infected died between 10 and 15 days post-infection, with evidence of delayed virus replication relative to KFDV-infected mice. The course of KFDV infection closely resembles the disease seen following infection with the prototypic tick-borne hemorrhagic flavivirus, OHFV, whereas AHFV-infected mice displayed less severe clinical disease more similar to the encephalitic flaviviruses [21], [22]. AHFV and KFDV, along with OHFV, are categorized as BSL-4 viruses and are on the Select Agent list of potential biothreat agents and are considered to be the only tick-borne flaviviruses that cause hemorrhagic manifestations in people. These features emphasize the importance of a reliable animal model for these severe pathogens. In the models described here, KFDV and AHFV infected mice develop disease similar to that seen in humans, providing an excellent platform for testing promising vaccines, antivirals and therapeutics.

## References

[1] Pattnaik P (2006) Kyasanur forest disease: an epidemiological view in India. Rev Med Virol 16: 151–165 10.1002/rmv.495 16710839

[2] Madani TA, Azhar EI, Abuelzein E-TME, Kao M, Al-Bar HMS, et al (2010) Alkhumra (Alkhurma) virus outbreak in Najran, Saudi Arabia: Epidemiological, clinical, and Laboratory characteristics. J Infect. 10.1016/j.jinf.2010.09.032 20920527

[3] Alzahrani AG, Shaiban Al HM, Mazroa Al MA, Al-Hayani O, Macneil A, et al (2010) Alkhurma hemorrhagic Fever in humans, najran, saudi arabia. Emerging Infectious Diseases 16: 1882–1888. 21122217 10.3201/eid1612.100417 PMC3294564

[4] Adhikari Prabha MR, Prabhu MG, Raghuveer CV, Bai M, Mala MA (1993) Clinical study of 100 cases of Kyasanur Forest disease with clinicopathological correlation. Indian J Med Sci 47: 124–130. 8225455

[5] Zaki AM (1997) Isolation of a flavivirus related to the tick-borne encephalitis complex from human cases in Saudi Arabia. Trans R Soc Trop Med Hyg 91: 179–181. 9196762 10.1016/s0035-9203(97)90215-7

[6] Memish ZA, Fagbo SF, Assiri AM, Rollin P, Zaki AM, et al (2012) Alkhurma viral hemorrhagic fever virus: proposed guidelines for detection, prevention, and control in Saudi Arabia. PLoS Negl Trop Dis 6: e1604 10.1371/journal.pntd.0001604 22860139 PMC3409106

[7] Dodd KA, Bird BH, Khristova ML, Albariño CG, Carroll SA, et al (2011) Ancient ancestry of KFDV and AHFV revealed by complete genome analyses of viruses isolated from ticks and mammalian hosts. PLoS Negl Trop Dis 5: e1352 10.1371/journal.pntd.0001352 21991403 PMC3186760

[8] Carletti F, Castilletti C, Di Caro A, Capobianchi MR, Nisii C, et al (2010) Alkhurma hemorrhagic Fever in travelers returning from egypt, 2010. Emerging Infectious Diseases 16: 1979–1982. 21122237 10.3201/eid1612101092 PMC3294557

[9] Charrel RN, Fagbo S, Moureau G, Alqahtani MH, Temmam S, et al (2007) Alkhurma hemorrhagic fever virus in Ornithodoros savignyi ticks. Emerging Infectious Diseases 13: 153–155. 17370534 10.3201/eid1301.061094 PMC2725816

[10] Mahdi M, Erickson BR, Comer JA, Nichol ST, Rollin PE, et al (2011) Kyasanur forest disease virus alkhurma subtype in ticks, najran province, saudi arabia. Emerging Infectious Diseases 17: 945–947. 21529425 10.3201/eid1705.101824 PMC3321790

[11] Kasabi GS, Murhekar MV, Yadav PD, Raghunandan R, Kiran SK, et al (2013) Kyasanur forest disease, India, 2011–2012. Emerging Infectious Diseases 19: 278–281 10.3201/eid1902.120544 23343570 PMC3559039

[12] Zivcec M, Safronetz D, Feldmann H (2013) Animal Models of Tick-Borne Hemorrhagic Fever Viruses. Pathogens 2: 402–421 10.3390/pathogens2020402 25437041 PMC4235721

[13] Carletti F (2010) Alkhurma Hemorrhagic Fever in Travelers Returning from Egypt, 2010. Emerging Infectious Diseases: 1–8. 10.3201/eid1612101092 21122237 PMC3294557

[14] Pavri K (1989) Clinical, clinicopathologic, and hematologic features of Kyasanur Forest disease. Reviews of Infectious Diseases 11 Suppl 4: S854–S859. 2665018 10.1093/clinids/11.supplement_4.s854

[15] Madani TA (2005) Alkhumra virus infection, a new viral hemorrhagic fever in Saudi Arabia. J Infect 51: 91–97 10.1016/j.jinf.2004.11.012 16038757

[16] Memish ZA, Charrel RN, Zaki AM, Fagbo SF (2010) Alkhurma haemorrhagic fever—a viral haemorrhagic disease unique to the Arabian Peninsula. International Journal of Antimicrobial Agents 36 Suppl 1: S53–S57 10.1016/j.ijantimicag.2010.06.022 20800999

[17] Kamphuis E, Junt T, Waibler Z, Forster R, Kalinke U (2006) Type I interferons directly regulate lymphocyte recirculation and cause transient blood lymphopenia. Blood 108: 3253–3261 10.1182/blood-2006-06-027599 16868248

[18] Memish ZA, Balkhy HH, Francis C, Cunningham G, Hajeer AH, et al (2005) Alkhumra haemorrhagic fever: case report and infection control details. Br J Biomed Sci 62: 37–39. 15816213 10.1080/09674845.2005.11978070

[19] Kimura T, Sasaki M, Okumura M, Kim E, Sawa H (2010) Flavivirus encephalitis: pathological aspects of mouse and other animal models. Vet Pathol 47: 806–818 10.1177/0300985810372507 20551474

[20] Muñoz-Jordán JL, Fredericksen BL (2010) How flaviviruses activate and suppress the interferon response. Viruses 2: 676–691 10.3390/v2020676 21994652 PMC3185611

[21] Tigabu B, Juelich T, Holbrook MR (2010) Comparative analysis of immune responses to Russian spring-summer encephalitis and Omsk hemorrhagic fever viruses in mouse models. Virology 408: 57–63 10.1016/j.virol.2010.08.021 20875909 PMC2966520

[22] Holbrook MR, Aronson JF, Campbell GA, Jones S, Feldmann H, et al (2005) An animal model for the tickborne flavivirus—Omsk hemorrhagic fever virus. The Journal of Infectious Diseases 191: 100–108 10.1086/426397 15593010

